# Effects of Consuming Beverages Sweetened with Fructose, Glucose, High-Fructose Corn Syrup, Sucrose, or Aspartame on OGTT-Derived Indices of Insulin Sensitivity in Young Adults

**DOI:** 10.3390/nu16010151

**Published:** 2024-01-02

**Authors:** Bettina Hieronimus, Valentina Medici, Vivien Lee, Marinelle V. Nunez, Desiree M. Sigala, Andrew A. Bremer, Chad L. Cox, Nancy L. Keim, Jean-Marc Schwarz, Giovanni Pacini, Andrea Tura, Peter J. Havel, Kimber L. Stanhope

**Affiliations:** 1 Department of Molecular Biosciences, School of Veterinary Medicine, University of California, Davis, CA 95616, USA; bettina.hieronimus@mri.bund.de (B.H.);; 2 Department of Physiology and Biochemistry of Nutrition, Max Rubner-Institut, 76131 Karlsruhe, Germany; 3 Division of Gastroenterology and Hepatology, University of California, Davis, CA 95616, USA; 4 Department of Nutrition, University of California, Davis, CA 95616, USA; 5 Department of Pediatrics, School of Medicine, University of California, Davis, CA 95616, USA; 6 Department of Chemistry and Department of Family and Consumer Sciences, California State University, Sacramento, CA 95819, USA; 7 United States Department of Agriculture, Western Human Nutrition Research Center, Davis, CA 95819, USA; 8 Department of Basic Sciences, College of Osteopathic Medicine, Touro University California, Vallejo, CA 94592, USA; 9 Department of Medicine, Division of Endocrinology, Zuckerberg San Francisco General Hospital, University of California San Francisco, San Francisco, CA 94110, USA; 10 Consiglio Nazionale delle Ricerche, Institute of Neuroscience, I-35121 Padova, Italy

**Keywords:** dietary intervention study, fructose, glucose, high-fructose corn syrup, sucrose, aspartame, insulin sensitivity index, insulin resistance, hepatic insulin sensitivity, muscle insulin sensitivity

## Abstract

(1) Background: Clinical results on the effects of excess sugar consumption on insulin sensitivity are conflicting, possibly due to differences in sugar type and the insulin sensitivity index (ISI) assessed. Therefore, we compared the effects of consuming four different sugars on insulin sensitivity indices derived from oral glucose tolerance tests (OGTT). (2) Methods: Young adults consumed fructose-, glucose-, high-fructose corn syrup (HFCS)-, sucrose-, or aspartame-sweetened beverages (SB) for 2 weeks. Participants underwent OGTT before and at the end of the intervention. Fasting glucose and insulin, Homeostatic Model Assessment-Insulin Resistance (HOMA-IR), glucose and insulin area under the curve, Surrogate Hepatic Insulin Resistance Index, Matsuda ISI, Predicted M ISI, and Stumvoll Index were assessed. Outcomes were analyzed to determine: (1) effects of the five SB; (2) effects of the proportions of fructose and glucose in all SB. (3) Results: Fructose-SB and the fructose component in mixed sugars negatively affected outcomes that assess hepatic insulin sensitivity, while glucose did not. The effects of glucose-SB and the glucose component in mixed sugar on muscle insulin sensitivity were more negative than those of fructose. (4) Conclusion: the effects of consuming sugar-SB on insulin sensitivity varied depending on type of sugar and ISI index because outcomes assessing hepatic insulin sensitivity were negatively affected by fructose, and outcomes assessing muscle insulin sensitivity were more negatively affected by glucose.

## 1. Introduction

Approximately 1 in 10 or over 500 million adults worldwide have diabetes, with individuals with type 2 diabetes mellitus (T2DM) representing over 90% of cases [1]. Insulin resistance, the impaired biological response of target tissues to insulin stimulation, is considered a central [2] or pivotal pathogenic component [3] of T2DM and metabolic syndrome. While criteria to diagnose the presence of insulin resistance have not been well defined, insulin sensitivity is assessed through numerous indices. These include static indices, which are easily assessed because they are based on single measurements of fasting circulating glucose and insulin concentrations. Assessing the dynamic indices based on circulating glucose and insulin concentrations during OGTT is more challenging. However, these OGTT-derived indices, which include Surrogate Hepatic Insulin Resistance Index (IRI) [4], Matsuda ISI [5], Predicted M ISI [6], and Stumvoll Index [7], are easier to obtain compared with the ISI derived from hyperinsulinemic euglycemic clamp, which is considered the gold standard for assessing insulin sensitivity [8].

Sugar-sweetened beverages (SSB) are a risk factor for weight gain and T2DM [9]. Observational studies have demonstrated positive associations between increased consumption of SSB and insulin resistance [10], T2DM [11], and metabolic syndrome [12]. Dietary intervention studies have shown that increased and sustained (>6 days) consumption of fructose-SB decreased hepatic insulin sensitivity [13,14,15], and increased OGTT glucose and insulin responses [16] as well as HOMA-IR [17]. Previous reports from this study showed consuming sucrose- or HFCS-SB negatively affected Matsuda ISI, Predicted M ISI, and glucose and insulin OGTT responses [18,19]. In contrast, a 6-month clinical trial found no effects of sucrose-SB consumption on OGTT responses [20]. Conflicting results also exist for glucose-SB, which have been shown to have either no effects [16] or negative effects on insulin sensitivity [14,21].

The inconsistent results may relate to the heterogeneity of the study protocols with regard to the types and doses of sugars studied and the outcomes used to assess insulin sensitivity. Therefore, the objectives of this study are to compare consumption of beverages sweetened with fructose, glucose, HFCS, or sucrose at 25% energy requirement (Ereq), or with aspartame on OGTT-derived static and dynamic markers of glucose tolerance and insulin sensitivity, and to determine the relative contribution of fructose and glucose in mediating the changes in each outcome.

## 2. Materials and Methods

### 2.1. Previous Publications

In this 5-year investigation, 187 participants were assigned to eight dietary interventions in which they consumed beverages sweetened with 25% Ereq fructose, 25% Ereq HFCS, 25% Ereq sucrose, 25% Ereq glucose, 17.5% Ereq fructose, 17.5% Ereq HFCS, 10% Ereq HFCS, or 0% Ereq aspartame. Previous reports from this study comparing metabolic outcomes between beverage groups have been published [18,19,22,23,24,25,26], including papers comparing indices of insulin sensitivity after consumption of 0 (aspartame-SB), 10, 17.5, or 25% of Ereq as HFCS-SB [18], and after consumption of 0 (aspartame-SB), or 25% Ereq as HFCS- or sucrose-SB [19]. To compare the effects of all major sugars on indices of insulin sensitivity, and effects of the proportions of fructose and glucose in all SB, the data from participants consuming 25% Ereq as HFCS- and sucrose-SB and from the control group (0% Ereq as aspartame-SB) were included in the current analyses.

### 2.2. Participants

The current paper reports the results from four of the experimental groups that consumed beverages sweetened with 25% Ereq as HFCS (*n* = 28), fructose (*n* = 28), glucose (*n* = 28), or sucrose (*n* = 24), and from the control group that consumed 0% Ereq as aspartame (*n* = 23) for 16 days. The CONSORT Chart (Supplement Figure S1) details the number of participants allocated to each of the five groups (overall: 144), the number who received intervention beverages (overall: 138), and the number included in analyses (overall: 131), yielding a retention rate of 91%.

The study protocol was approved by the University of California, Davis Institutional Review Board and is registered with clinicaltrials.gov: NCT01103921. Participants were recruited between October 2008 and February 2014 through an internet listing (https://Craigslist.com; accessed on 25 December 2023) and local posting of flyers. Pre-screening was conducted via telephone, and consenting and collection of a fasting blood sample were conducted during a 1 h in-person interview. Inclusion criteria included body mass index (BMI) 18–35 kg/m^2^, age 18–40 years, and stable body weight (self-reported) during the prior six months. Individuals exhibiting evidence of disease including blood pressure > 160/90 mmHg, fasting glucose ≥ 6.0 mmol/L, and fasting triglyceride > 4.5 mmol/L were excluded. Individuals who reported weight loss surgery, smoking, consuming >2 servings of sugar-SB or alcoholic beverages/day, exercising >3.5 h/day at a level more vigorous than walking, and use of medications to lower or treat lipids, glucose, blood pressure, body weight, depression, or thyroid conditions were also excluded. Prior to enrollment, all participants provided written informed consent. For up to 5 weeks before the start of study, scheduled participants were asked to avoid all sugar-containing drinks, except unsweetened milk and one 8 oz serving of 100% fruit juice per day, and to stop using any dietary supplements.

### 2.3. Study Protocol

**Figure 1:**
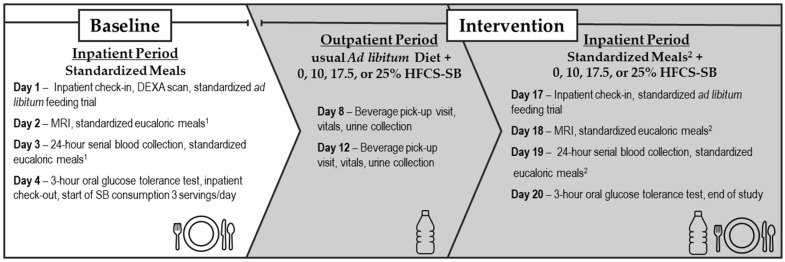
Study protocol.

This was a parallel-arm, double-blinded dietary intervention study. The experimental groups were matched for BMI, sex, and fasting serum concentrations of insulin, low-density and high-density lipoprotein cholesterol, and triglyceride. As shown in Figure 1, the study design consisted of three phases: (1) An inpatient baseline period lasting 3.5 days at the University of California Davis Clinical and Translational Science Center’s Clinical Research Center (CCRC). Participants consumed a standardized low-sugar diet and baseline experimental procedures were conducted. (2) An outpatient intervention period lasting 12.5 days. Participants consumed their assigned intervention beverages while maintaining their usual dietary and exercise habits. (3) An inpatient intervention period lasting 3.5 days at the CCRC. Participants consumed standardized low-sugar meals with their assigned intervention beverages and intervention experimental procedures were conducted.

#### 2.3.1. Inpatient Meals

On Study Days 1 (baseline) and 17 (intervention) fasting body weight was measured and standardized low sugar meals were provided ad libitum [23]. On inpatient Study Days 2 and 3 (baseline), and 18 and 19 (intervention), participants consumed energy-balanced meals consisting of 55% Ereq carbohydrate, 30% fat, and 15% protein. During baseline meals, the carbohydrate consisted primarily of low-fiber complex carbohydrate. The meals provided during the intervention were identical to baseline meals, except for the isocaloric replacement of complex carbohydrate with the sugar in the assigned beverages. Breakfast, lunch, and dinner were served at 9:00, 13:00, and 18:00 h and consisted of 25%, 35%, and 40% of daily Ereq, respectively. The Mifflin equation, with an activity adjustment of 1.3 during the 24-h blood collections and of 1.5 for the other inpatient days [27], was used to calculate daily Ereq.

#### 2.3.2. Study Beverages and Outpatient Meals

The sugar-SB contained 25% Ereq as glucose (STALEYDEX^®^, Tate & Lyle, Hoffman Estates, IL, USA), fructose (KRYSTAR^®^, Tate & Lyle), HFCS (ISOSWEET^®^ 5500, 55% fructose, 45% glucose, Tate & Lyle), or sucrose (C&H^®^ cane sugar, Domino Foods Inc., Yonkers, NJ, USA), and were flavored with unsweetened Kool-Aid^®^ drink mix. A fruit-flavored aspartame drink mix (Market Pantry^®^, Milwaukee, WI, USA) was used to prepare the 0% Ereq beverages. Participants and study personnel who interacted with participants or analyzed samples were blinded to beverage assignment. The grams of beverage provided to each subject was based on their calculated Ereq. During the 12-day outpatient phase, participants were instructed to drink three study beverages/day, one/meal; to consume their usual diet ad libitum; and to not consume any other sweet beverages, including fruit juice. Riboflavin was added to all beverages as a compliance marker. Participants were informed of the presence of a biomarker but not its identity. Spot urine was collected during inpatient days and during the three beverage pick-up visits. Urinary riboflavin was measured fluorometrically, and urinary riboflavin concentrations suggested good compliance in all groups [22,23].

#### 2.3.3. Insulin Sensitivity

Oral Glucose Tolerance Tests (OGTT) were performed on Study Day 4 (baseline: Week 0) and Study Day 20 (intervention: Week 2). Blood samples were collected through an intravenous catheter before (8:00 h) and 30, 60, 90, 120, and 180 min after consumption of a 75 g glucose beverage. Plasma glucose concentrations were analyzed using a YSI glucose analyzer (YSI Inc., Yellow Springs, OH, USA), and the intra- and inter-assay CVs were 3.6% and 4.5%, respectively. Plasma insulin concentrations were analyzed via radioimmunoassay (Millipore Inc., St. Charles, MO, USA), and the intra- and inter-assay CVs were 6.5% and 7.6%, respectively. Fasting glucose and insulin data were used to calculate HOMA-IR [28] and QUICKI [29]. Three-hour OGTT data were used to calculate total area under the curve (AUC) for glucose and insulin via the trapezoidal method. Fasting and 30 min values were used to calculate the Surrogate Hepatic Insulin Resistance Index (IRI) [4]. Two-hour OGTT data were used to derive the Matsuda ISI [5], Predicted M ISI [6], and Stumvoll Index [7]. Due to missing OGTT samples, Predicted M and Stumvoll Index could not be calculated for three participants (aspartame-SB = 1; 25% HFCS-SB = 1; 25% fructose-SB = 1).

#### 2.3.4. Outpatient 24-h Food Intake Recalls

As detailed in the Supplement (S1.2), 24-h food intake recalls during the outpatient intervention period were collected using the USDA 5-step Multiple-Pass Method [30] or the Automated Self-Administered (ASA) 24-h Dietary Assessment Tool (https://epi.grants.cancer.gov/asa24/, accessed on 28 December 2023).

### 2.4. Statistical Analyses

Tested outcomes were the absolute change (∆) at 2 weeks of intervention compared with the baseline. Outcomes were log transformed when baseline or absolute ∆ values were not normally distributed (Shapiro–Wilk). Effects of the five groups on each outcome were tested in a two-factor (beverage, sex) general linear model (GLM) analysis of covariance (ANCOVA) (SAS 9.3; SAS, Cary, NC, USA) with adjustment for metabolic syndrome risk factors (MSRF) and outcome at baseline. MSRF scores at baseline were assessed through assigning a value of one to measured clinical outcomes outside of standard established cutoffs as previously described [31]. Significant differences between groups were identified using Tukey’s multiple comparisons test. Significant changes from baseline within intervention groups were identified as least squares mean (LS mean) of ∆ different from zero. In additional analyses, the same model included adjustment for Δ body weight.

Log transformation improved but did not normalize the Stumvoll Index. Therefore, the beverage group effect was analyzed using the Kruskal–Wallis nonparametric test, and the within and between group differences were analyzed using the Wilcoxon matched-pairs signed rank test and the Mann–Whitney U test, respectively.

Post-hoc analyses were conducted to determine the relative effects of fructose and glucose on each ISI outcome. As previously described [22], the proportions of fructose and glucose in each of the five intervention beverages were described as separate variables, thus, respective to fructose and glucose, fructose-SB were inputted as 100 and 0, glucose-SB as 0 and 100, HFCS as 55 and 45, sucrose-SB as 50 and 50, and aspartame-SB as 0 and 0. Δ of each outcome was analyzed using GLM multivariable (fructose, glucose) ANCOVA with adjustment for sex, MSRF, and outcome at baseline. The interaction term fructose*glucose was tested in the initial models but removed as it did not improve the sensitivity of any of the analyses. The proportion of variance explained by the model and byf fructose and glucose was calculated as (type III sum of squares/corrected total sum of squares) × 100. The transformed values of the Stumvoll Index that provided the most normal distribution were used for these analyses. Statistical significance was considered at *p* < 0.05. Data are reported as mean ± standard deviation (SD) in Table 1 and mean ± standard error of the mean (SEM) for all others.

## 3. Results

### 3.1. Outcomes at Baseline

There were no differences between the five experimental groups in baseline anthropomorphic or metabolic parameters (Table 1).

**Table 1:** Participant characteristics at baseline.

Parameter	25% HFCS (*n* = 28)	25% Sucrose (*n* = 24)	25% Fructose (*n* = 28)	25% Glucose (*n* = 28)	Aspartame (*n* = 23)
Age (year)	26.8 ± 6.6	25.9 ± 6.1	26.8 ± 6.2	26.0 ± 5.7	25.4 ± 6.2
Sex (M/F)	15/13	12/12	15/13	15/13	11/12
BMI (kg/m^2^)	24.9 ± 4.0	25.3 ± 3.4	25.4 ± 3.7	25.8 ± 3.4	24.8 ± 3.3
Waist circumference (cm)	77.0 ± 10.1	75.4 ± 7.3	78.3 ± 10.2	79.0 ± 9.3	75.2 ± 6.4
Body fat (%)	26.0 ± 9.7	29.1 ± 11.3	29.0 ± 10.3	28.9 ± 8.4	27.1 ± 9.6
Energy requirement (kcal/d)	2390 ± 350	2351 ± 310	2450 ± 324	2431 ± 309	2354 ± 322
Systolic blood pressure (mm Hg)	117 ± 10	114 ± 8	117 ± 10	119 ± 11	112 ± 12
Diastolic blood pressure (mm Hg)	73 ± 7	72 ± 6	72 ± 7	74 ± 8	69 ± 9
Fasting triglyceride (mmol/L)	1.2 ± 0.6	1.3 ± 0.6	1.1 ± 0.4	1.1 ± 0.5	1.1 ± 0.6
Fasting total cholesterol (mmol/L)	4.1 ± 0.9	4.1 ± 0.6	3.9 ± 0.6	4.2 ± 0.8	3.9 ± 0.7
Fasting HDL cholesterol (mmol/L)	1.2 ± 0.4	1.1 ± 0.2	1.2 ± 0.2	1.2 ± 0.4	1.0 ± 0.2
MSRF	1.1 ± 1.0	0.9 ± 1.1	0.8 ± 0.9	1.0 ± 0.9	1.2 ± 0.7

Values are mean ± SD; HFCS, high-fructose corn syrup; MSRF, metabolic syndrome risk factor score.

### 3.2. Glucose and Insulin Responses during OGTT

**Figure 2:**
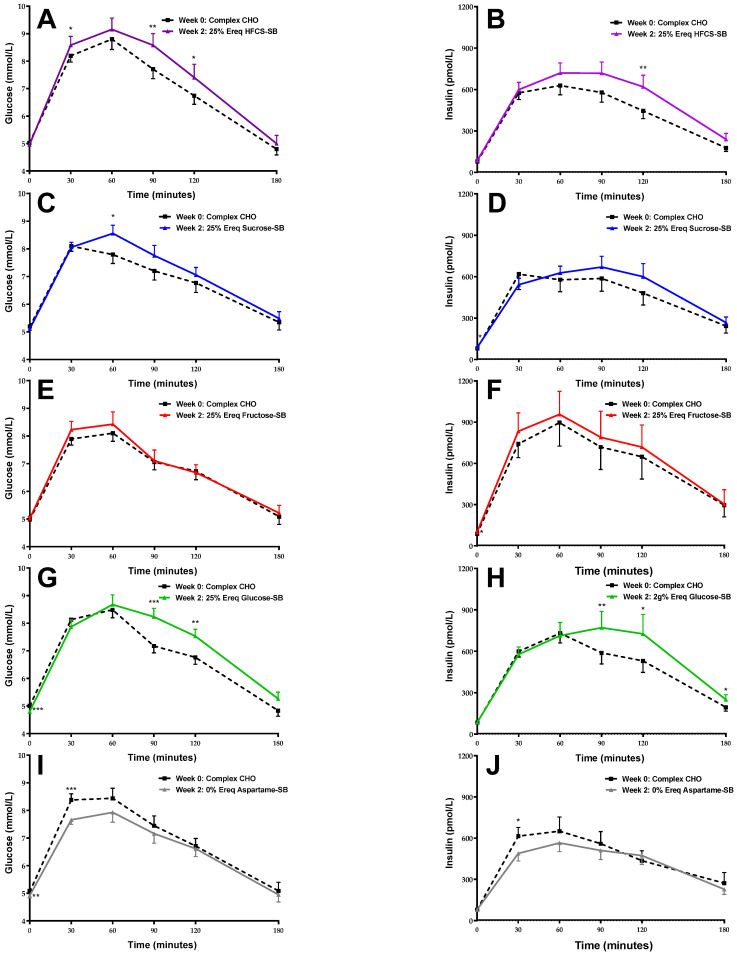
The group means of the OGTT glucose and insulin profiles at baseline (Week 0) and the end of intervention (Week 2) in participants consuming HFCS- ((**A**,**B**) respectively, *n* = 28), sucrose- ((**C**,**D**), *n* = 24), fructose- ((**E**,**F**), *n* = 28), glucose- ((**G**,**H**), *n* = 28), or aspartame-SB ((**I**,**J**), *n* = 23). * *p* < 0.05, ** *p* < 0.01, *** *p* < 0.001, paired *t* test.

Figure 2 shows the mean glucose and insulin responses at baseline (Week 0) and at the end of the intervention (Week 2) in participants consuming HFCS-, sucrose-, fructose-, glucose-, and aspartame-SB.

### 3.3. Body Weight and OGTT-Derived Outcomes

The group means for body weight and the OGTT-derived outcomes at Week 0 (baseline) and Week 2 (end of intervention), with P values for the effect of beverage and MSRF, are shown in Table 2.

### 3.4. Body Weight

Body weight was not significantly affected by beverage (Supplement Figure S2). Importantly, including adjustment for the change in body weight in secondary ANCOVAs showed that it had no effect on the OGTT-derived outcomes.

### 3.5. Static Indices and Surrogate Hepatic IRI

**Figure 3:**
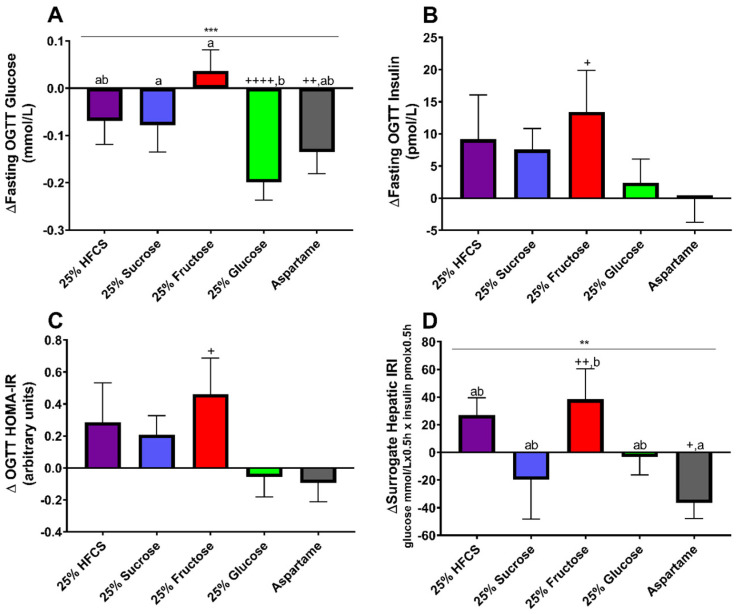
Δ fasting glucose (**A**), insulin (**B**), HOMA−IR (**C**), and Surrogate Hepatic IRI (**D**) (Week 2–Week 0) during OGTT in participants consuming beverages sweetened with HFCS−, sucrose−, fructose−, glucose−, or aspartame-sweetened beverages for 2 weeks. ** *p* < 0.01, *** *p* < 0.001, effect of beverage, two−factor (beverage, sex) ANCOVA with adjustment for MSRF and outcome at baseline. ^+^ *p* < 0.05, ^++^ *p* < 0.01, ^++++^ *p* < 0.0001, LS mean different from zero; groups without shared letters are significantly different, Tukey’s post-test.

Participants consuming glucose-SB exhibited decreased plasma concentrations of fasting glucose compared with the participants consuming fructose- (*p* = 0.0006) and sucrose-SB (*p* = 0.020) (Figure 3A). Fasting glucose was the only outcome affected by sex, with men exhibiting larger increases after the 2-week intervention than women (*p* < 0.0001). The changes in fasting insulin (Figure 3B), HOMA-IR (Figure 3C), and QUICKI (Supplement Figure S3) were not different between beverage groups and all three outcomes were detrimentally affected by MSRF. The Surrogate Hepatic IRI was significantly increased in participants consuming fructose-SB compared with those consuming aspartame-SB (*p* = 0.010) (Figure 3D).

### 3.6. OGTT Glucose and Insulin 3 h AUC

**Figure 4:**
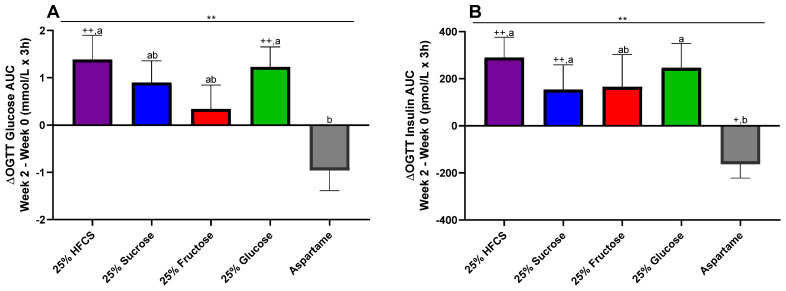
Δ 3 h glucose (**A**) and insulin (**B**) AUC (Week 2–Week 0) during OGTT in participants consuming beverages sweetened with HFCS-, sucrose-, fructose-, glucose-, or aspartame-sweetened beverages for 2 weeks. ** *p* < 0.01, effect of beverage, two-factor (beverage, sex) ANCOVA with adjustment for MSRF and outcome at baseline. ^+^ *p* < 0.05, ^++^ *p* < 0.01, LS mean different from zero; groups without shared letters are significantly different, Tukey’s post-test.

The OGTT glucose AUC was significantly increased in participants consuming glucose- or HFCS-SB compared with participants consuming aspartame-SB (glucose: *p* = 0.016; HFCS: *p* = 0.0067) (Figure 4A). The OGTT insulin AUC was significantly increased in participants consuming sucrose-, HFCS-, or glucose-SB compared with participants consuming aspartame-SB (sucrose: *p* = 0.0091; HFCS: *p* = 0.0092; glucose: *p* = 0.048) (Figure 4B).

### 3.7. Dynamic OGTT Indices

**Figure 5:**
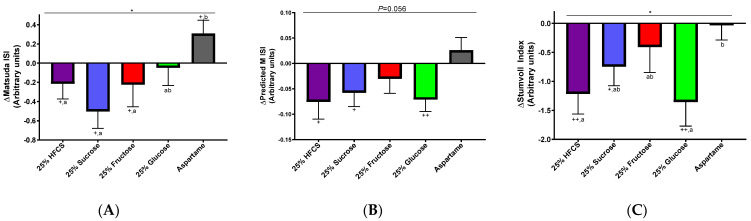
Absolute ΔMatsuda ISI (**A**), ΔPredicted M ISI (**B**), and ΔStumvoll Index (**C**) after young adults consume beverages sweetened with 25% Ereq HFCS, sucrose, fructose, or glucose; or with aspartame for 2 weeks. A and B: two-factor (beverage, sex) ANCOVA (SAS 9.4) with adjustment for MSRF and outcome at baseline. *p* = 0.056, * *p* < 0.05 effect of beverage; ^+^ *p* < 0.05, ^++^ *p* < 0.01 LS mean of change different than zero; groups without shared letters are significantly different, Tukey’s post-test. C: Kruskal–Wallis test, * *p* < 0.05 effect of beverage, ^+^ *p* < 0.05, ^++^ *p* < 0.01, Wilcoxon matched-pairs signed rank test of within-group 0 vs. 2 wk. Groups without shared letters are significantly different, Mann–Whitney U test.

The Matsuda ISI was significantly decreased by the consumption of fructose-, HFCS-, and sucrose-SB compared with the consumption of aspartame-SB (fructose: *p* = 0.036; HFCS: *p* = 0.018; sucrose: *p* = 0.0016) (Figure 5A). The changes in the Predicted M ISI were not significantly different among groups (*p* = 0.056) (Figure 5B). MSRF contributed to the decreases in the Matsuda and Predicted M ISI. The Stumvoll Index was decreased in participants consuming glucose-SB and HFCS-SB compared with participants consuming aspartame-SB (glucose: *p* = 0.0087; HFCS: *p* = 0.0064) (Figure 5C).

### 3.8. The Contributions of Fructose and Glucose

To investigate the relative contributions of fructose and glucose to the changes of the OGTT-derived outcomes, post-hoc analyses were performed in which the beverage groups were described as two independent variables based on their respective glucose and fructose content. Table 3 shows the % of variation explained by fructose, glucose, and the whole model for each outcome. Only fructose contributed to the increase of fasting glucose, HOMA-IR, and Hepatic IRI. Both fructose and glucose contributed to the increases of glucose and insulin OGTT 3 h AUC, with glucose having the greater effect, especially on the glucose AUC. Both sugars contributed to the decreases in the Matsuda and Predicted M ISI, with fructose having the greater effect on the Matsuda ISI and glucose having the greater effect on the Predicted M ISI. Only glucose contributed to the decrease in the Stumvoll ISI.

## 4. Discussion

### 4.1. Static Indices and Surrogate Hepatic IRI

The static ISIs are considered to primarily reflect hepatic insulin sensitivity, specifically the capacity of insulin to suppress hepatic glucose production in the fasting state [32]. Interestingly, consumption of glucose-SB lowered fasting glucose compared with fructose-SB, a finding also observed in our 10-week study comparing consumption of 25% Ereq fructose- with glucose-SB in older participants [16]. In the current study, fructose-SB was the only beverage group that increased the Surrogate Hepatic IRI compared with aspartame-SB. The Surrogate Hepatic IRI reflects fasting hepatic glucose production and the suppression of hepatic glucose production during the first 30 min of the OGTT [4]. The surrogate Hepatic IRI had a stronger correlation with hepatic glucose production during euglycemic insulin clamp than HOMA-IR [4]. Accordingly, the Surrogate Hepatic IRI was more sensitive than HOMA-IR or fasting insulin in detecting an effect of beverage group on the change of hepatic insulin sensitivity. The multivariable model comparing the relative effects of fructose and glucose showed that fructose explained more than tenfold % variation in hepatic insulin sensitivity indices than glucose. Neither sucrose- nor HFCS-SB affected these indices, suggesting that the exchange of 45–50% of the fructose for glucose dilutes their detrimental effects on hepatic insulin sensitivity. Overall, our results support findings from previous intervention trials in which fructose-SB decreased hepatic insulin sensitivity, assessed via euglycemic-hyperinsulinemic clamps, compared with glucose-SB [13] and complex carbohydrate [15].

The differential effects of fructose and glucose on hepatic insulin sensitivity are explained through the enzymatic activity controlling their hepatic metabolism. Phosphofructokinase regulates glycolysis based on liver energy status. In the energy-replete liver, glycolysis is blocked, allowing glucose to bypass the liver. Fructokinase, which controls fructose metabolism, is not affected by liver energy status, and remains active. As a result, the liver can take up as much as 85% of a large fructose dose [33]. This exposure to fructose may directly impair insulin signaling [34]. However, hepatic fructose overload also causes upregulated de novo lipogenesis and impaired fat oxidation, increased hepatic fat accumulation, hepatic inflammation, and endoplasmic reticulum stress, and increased production of uric acid, reactive oxygen species [34], and lactic acid. One or several of these downstream effects may contribute to the detrimental effects of fructose on hepatic insulin sensitivity.

### 4.2. OGTT Glucose and Insulin AUC

Fructose-SB had less of an effect on OGTT glucose and insulin AUC than glucose-, HFCS-, and sucrose-SB. The OGTT glucose AUC provides an estimate of glucose tolerance [35], or more specifically, impaired glucose tolerance, which is associated with muscle insulin resistance [36]. The OGTT insulin AUC increases with insulin resistance due to both the increased need for insulin to compensate for impaired insulin action and reduced insulin clearance mainly by the liver [8]. The multivariable model showed that both glucose and fructose contributed to increased glucose and insulin AUC, with glucose accounting for more of the % variation.

### 4.3. Dynamic Indices

It is considered a major disadvantage of static indices that they fail to provide information about muscle insulin sensitivity [32]. Therefore, several research groups have developed dynamic ISIs through fitting OGTT data to data obtained during hyperinsulinemic euglycemic clamps in the same subjects. We examined the effects of sweetened beverages on two of the most utilized of these indices [8]: Matsuda ISI [5] and Stumvoll Index [7]; and on a more recently developed index, Predicted M ISI [6].

While HFCS-SB and sucrose-SB, which contain both fructose and glucose, had consistent detrimental effects on these indices, the effects of fructose- and glucose-SB were not consistent. Consumption of glucose-SB decreased the Predicted M ISI and the Stumvoll Index and fructose-SB did not, while consumption of fructose-SB decreased the Matsuda ISI and glucose-SB did not. The multivariable model comparing the effects of fructose and glucose showed both contributed to the decreases in the Matsuda ISI and the Predicted M ISI, with fructose having the stronger effect on the Matsuda ISI and glucose having the stronger effect on the Predicted M ISI. Only glucose contributed to the decrease in the Stumvoll Index. These disparate effects are likely due to the extent to which each index assesses muscle insulin sensitivity versus a combination of hepatic and muscle insulin sensitivity. The Matsuda ISI was developed as a composite index that reflects both hepatic and muscle insulin sensitivity [5]. Therefore, the greater effect of fructose compared to glucose on the Matsuda ISI can be explained by the hepatic insulin sensitivity component of the index. The Predicted M ISI was modeled to be comparable to the clamp-derived M value (glucose infused during steady-state period [6]). The Stumvoll Index was modeled on the glucose clearance rate during clamp procedures [7]. Both the Predicted M ISI and the Stumvoll Index, therefore, serve as markers of muscle insulin sensitivity, as the glucose infused and cleared during the steady state is primarily utilized by muscle. Thus, our current results, in which glucose had a greater effect on the Predicted M ISI and the Stumvoll Index than fructose, suggest that glucose consumption had a more detrimental effect on muscle insulin sensitivity than fructose consumption, and that it is the glucose component of sucrose and HFCS that predominantly mediated the decreases in muscle insulin sensitivity that were observed when these sugars were consumed.

However, these are unexpected findings as they differ from our previous intervention study where we compared the effects of consuming 25% Ereq fructose- and glucose-SB [16]. In that study, we found that consuming fructose-SB resulted in larger increases in the 3 h OGTT glucose and insulin AUCs, and a significant decrease in the deuterated glucose disposal insulin sensitivity index compared to glucose-SB consumption [16]. In order to compare the changes in muscle insulin sensitivity between the two studies using the same ISI, we calculated the Stumvoll Index for the previous study, post hoc. The Stumvoll Index was decreased in participants consuming fructose (−1.6 ± 0.5 mg/min/kg, *p* = 0.0003 Wilcoxon matched-pairs signed rank test) but not in participants consuming glucose (−0.3 ± 0.5 mg/min/kg, *p* = 0.61). The contrasting results between the current and previous [16] study could possibly be explained by the length of sugar exposure (current study: 2 weeks; previous study: 10 weeks) and/or by the difference in participant characteristics (current study: age 26 ± 6 years and BMI 25.3 ± 3.5; previous study: age 54 ± 8 years and BMI 29.3 ± 2.9 kg/m^2^).

It is likely that the mechanisms by which fructose and glucose impair muscle insulin sensitivity are different because their exposure to muscle is different. Since dietary fructose is metabolized mainly in the liver, circulating fructose levels are low after consumption [37]. Thus, the mechanism by which dietary fructose impairs muscle insulin sensitivity likely involves a mediator such as intramyocellular lipid, lactate, or uric acid [18,38].

In contrast to fructose, consumption of glucose increases circulating glucose and insulin levels [39]. Both hyperglycemia and hyperinsulinemia could be involved in the detrimental effects of glucose consumption on muscle insulin sensitivity. There is evidence from in vitro and animal studies showing that hyperglycemia has deleterious effects on insulin signaling in muscle [40]. Recent results indicate that hyperinsulinemia reduces insulin receptor gene expression in muscle [41].

Interestingly, both the Surrogate Hepatic IRI and the Matsuda ISI appeared to be beneficially affected by consumption of aspartame-SB. It is unlikely these positive effects were mediated via a direct metabolic effect of aspartame. Instead, they could have occurred because participants eliminated their usual consumption of sugar-SB during the study as the dietary protocol prohibited the consumption of any self-selected sugar-containing beverages. Several studies have demonstrated that lowering consumption of sugar-SB and/or all sugar-sweetened foods leads to positive health effects [42,43].

### 4.4. Study Strengths and Limitations

The OGTT were preceded by 3-day inpatient periods during which participants lived at the CCRC and consumed standardized low-sugar meals. Exercise consisted of two 20 minute staff-supervised walks during each 3-day period. Thus, the acute effects that variable diets and physical activity levels could have on OGTT results were eliminated, and this may explain why this study detected effects of consuming sugar-sweetened beverages on OGTT-derived indices that one study lasting 6 months did not [20]. The biomarker (riboflavin) in the beverages provided an objective measure of compliance, which was generally good as evidenced by the consistent dose–response trends we have previously reported [18,25].

A limitation of the study was that it was not randomized, thus potentially introducing unintentional bias in the assignment of participants to the experimental groups. During the 12-day outpatient period, participants consumed the study beverages with ad libitum diets of their choice, therefore, the total amount of sugar and other dietary components consumed during this period cannot be accurately quantified. However, the 24-h food records collected during the intervention suggest that the outpatient diet was not different among the subjects consuming HFCS-, sucrose-, fructose-, or glucose-SB (See Supplement Tables S1 and S2). The duration of the 2-week intervention is relatively short, which could also be considered a limitation. However, it demonstrates how quickly excess consumption of sugar-sweetened beverages can initiate metabolic dysregulation.

## 5. Conclusions

Consumption of sugar-SB affected glucose tolerance and insulin sensitivity; however, the effects differed depending on the type of sugar consumed and the ISI utilized. Outcomes that mainly reflect hepatic insulin sensitivity (fasting insulin, HOMA-IR, Surrogate Hepatic IRI, and Matsuda ISI) were detrimentally affected by consumption of fructose-SB. In contrast, outcomes that reflect glucose tolerance and muscle insulin sensitivity (OGTT glucose and insulin AUC, Predicted M ISI, and Stumvoll Index) were detrimentally affected by glucose-SB. Sucrose- and/or HFCS-SB had detrimental effects on Matsuda ISI, OGTT glucose and insulin AUC, Predicted M ISI, and Stumvoll Index. Analyses that elucidated the effects of the proportion of fructose and glucose in all beverages confirmed that only fructose decreased hepatic insulin sensitivity and showed that both fructose and glucose promoted detrimental effects on muscle insulin sensitivity; however, the effects of glucose were more prominent. Our finding that glucose had more potent effects on muscle insulin sensitivity than fructose needs to be confirmed in studies with longer duration and with more diverse populations. 

## Figures and Tables

**Table 2:** Outcomes (Week 0) before and after intervention (Week 2).

Outcome	25% HFCS	25% Sucrose	25% Fructose	25% Glucose	Aspartame	Effect of	*p*-Value
	(*n* = 28)	(*n* = 24)	(*n* = 28)	(*n* = 28)	(*n* = 23)		
Body Weight (kg)							
Week 0	72.9 ± 2.7	71.9 ± 2.4	75.7 ± 2.4	75.5 ± 2.4	71.8 ± 2.2	Beverage ^1^	0.097
Week 2	73.7 ± 2.8	72.4 ± 2.5	75.8 ± 2.4	76.1 ± 2.5	71.7 ± 2.2	MSRF ^2^	0.8
Fasting Glucose (mmol)							
Week 0	5.0 ± 0.1	5.2 ± 0.1	5.0 ± 0.1	5.0 ± 0.1	5.1 ± 0.1	Beverage	0.0009
Week 2	5.0 ± 0.1	5.1 ± 0.1	5.0 ± 0.1	4.8 ± 0.0	4.9 ± 0.1	MSRF	0.18
Fasting Insulin (pmol)							
Week 0	91.5 ± 6.1	92.5 ± 7.0	101.7 ± 11.4	96.8 ± 6.5	91.3 ± 7.2	Beverage	0.43
Week 2	100.6 ± 9.1	100.1 ± 7.7	115.1 ± 15.4	99.2 ± 6.7	91.2 ± 7.4	MSRF	0.016
HOMA-IR (arbitrary units)							
Week 0	3.0 ± 0.2	3.1 ± 0.2	3.3 ± 0.4	3.1 ± 0.2	3.0 ± 0.2	Beverage	0.19
Week 2	3.3 ± 0.3	3.3 ± 0.3	3.7 ± 0.5	3.1 ± 0.2	2.9 ± 0.2	MSRF	0.012
Surrogate Hepatic IRI (glucose mmol/L × 0.5 h × insulin pmol × 0.5 h)							
Week 0	97.7 ± 11.9	112.9 ± 34.6	130.8 ± 23.8	106.9 ± 13.1	116.9 ± 15.6	Beverage	0.009
Week 2	124.7 ± 17.1	93.1 ± 12.2	169.2 ± 36.8	103.4 ± 15.1	80.3 ± 14.6	MSRF	0.28
OGTT Glucose AUC (mmol/L × 3 h)							
Week 0	21.1 ± 0.7	20.6 ± 0.7	20.1 ± 0.6	20.6 ± 0.5	20.9 ± 0.7	Beverage	0.006
Week 2	22.5 ± 0.9	21.5 ± 0.5	20.4 ± 0.8	21.9 ± 0.6	19.9 ± 0.7	MSRF	0.53
OGTT Insulin AUC (pmol/L × 3 h)							
Week 0	1335 ± 106	1394 ± 212	1821 ± 370	1476 ± 154	1392 ± 184	Beverage	0.007
Week 2	1625 ± 154	1548 ± 161	1987 ± 392	1723 ± 228	1229 ± 151	MSRF	0.24
Matsuda ISI (arbitrary units)							
Week 0	3.3 ± 0.2	3.6 ± 0.3	3.3 ± 0.3	3.2 ± 0.2	3.6 ± 0.3	Beverage	0.01
Week 2	3.0 ± 0.3	3.1 ± 0.3	3.1 ± 0.3	3.1 ± 0.3	3.9 ± 0.3	MSRF	0.041
Predicted M ISI (arbitrary units)							
Week 0	1.52 ± 0.05	1.48 ± 0.06	1.46 ± 0.07	1.46 ± 0.05	1.52 ± 0.06	Beverage	0.056
Week 2	1.45 ± 0.06	1.42 ± 0.06	1.43 ± 0.07	1.39 ± 0.05	1.55 ± 0.05	MSRF	0.022
Stumvoll ISI (arbitrary units)							
Week 0	7.6 ± 0.5	7.5 ± 0.6	6.5 ± 1.0	7.1 ± 0.5	7.7 ± 0.5	Beverage ^3^	0.036
Week 2	6.4 ± 0.6	6.8 ± 0.6	6.1 ± 1.0	5.8 ± 0.8	7.7 ± 0.5	MSRF	…

Values are mean ± SEM; ^1^ effect of beverage in two-factor (beverage, sex) ANCOVA with adjustment for MSRF and outcome at baseline; ^2^ effect of MSRF in same model; ^3^ effect of beverage in Kruskal–Wallis nonparametric test. ANCOVA, analysis of covariance; HFCS, high-fructose corn syrup; MSRF, metabolic syndrome risk factor score; AUC, area under the curve.

**Table 3:** The relative contribution of fructose and glucose to the changes in OGTT-derived outcomes.

	Fructose		Glucose		Model ^1^	
Outcome	Variation (%) ^2^	*p*-Value	Variation (%)	*p*-Value	Variation (%)	*p*-Value
Fasting glucose	5	0.0034	0.075	0.25	29.7	<0.0001
Fasting insulin	2.6	0.059	0.27	0.47	10.1	0.02
HOMA-IR	2.7	0.024	0.11	0.59	11.7	0.0076
Surrogate Hepatic IRI	6.4	0.002	0.52	0.37	19.5	<0.0001
OGTT glucose AUC	3.8	0.021	9.4	0.0004	12.0	0.0065
OGTT insulin AUC	6.3	0.0031	7.8	0.001	13.3	0.0029
Matsuda ISI	8.3	0.0007	4.8	0.0098	10.3	0.0039
Predicted M ISI	3.4	0.0333	6.5	0.0033	11.2	0.012
Stumvoll ISI	1.1	0.24	5.8	0.0062	8.3	0.057

^1^ Multivariable (fructose, glucose) ANCOVA of absolute change of outcome with adjustment for sex, MSRF, and outcome at baseline; ^2^ calculated as (Sum of Squares/Corrected Total Sum of Squares) × 100.

## Data Availability

Restrictions apply to the availability of some or all data generated or analyzed during this study to preserve subject confidentially. The corresponding authors will on request detail the restrictions and any conditions under which access to some data may be provided.

## Supplementary Materials

The following supporting information can be downloaded at https://www.mdpi.com/article/10.3390/nu16010151/s1: Methods: Outpatient 24-h food intake recalls; Figure S1: Number of participants allocated, lost to follow-up and included in analyses in the groups consumincaspartame-, and 25% energy requirement glucose-, HFCS-, sucrose- and fructose-SB; Figure S2: A Body weight (Week 2–Week 0) in subjects consuming beverages sweetenedwith HFCS-, sucrose-, fructose-, glucose- or aspartame-sweetened beverages for 2 weeks(F). 2-factor (beverage, sex) ANCOVA with adjustment for MSRF and outcome at baseline. ^+^ *p* < 0.05, ^++^ *p* < 0.01, LS mean different from zero; Figure S3: Δ Quicki (Week 2–Week 0) in subjects consuming beverages sweetened withHFCS-, sucrose-, fructose-, glucose- or aspartame-sweetened beverages for 2 weeks (F). 2factor (beverage, sex) ANCOVA with adjustment for MSRF and outcome at baseline, ^+^ *p* < 0.05, ^++^ *p* < 0.01, LS mean different from zero; Table S1. 24-h recall energy intake during outpatient; Table S2: 24-h recall macronutrient intake during outpatient intervention.
