# Supplementary material for: Effects of Consuming Beverages Sweetened with Fructose, Glucose, High-Fructose Corn Syrup, Sucrose, or Aspartame on OGTT-Derived Indices of Insulin Sensitivity in Young Adults
Source: Nutrients. 2024 Jan 2;16(1):151. doi: 10.3390/nu16010151 (PMC10780640; doi:10.3390/nu16010151)
Supplement: Supplementary file 1 [file nutrients-16-00151-s001.zip › nutrients-2740514-supplementary.pdf]

Comparing the Effects of Consuming Beverages Sweetened with Fructose, Glucose, High Fructose Corn Syrup, Sucrose or Aspartame on OGTT-derived Indices of Insulin Sensitivity in Young Adults: Supplement

S1. Methods

S1.1 Consort chart

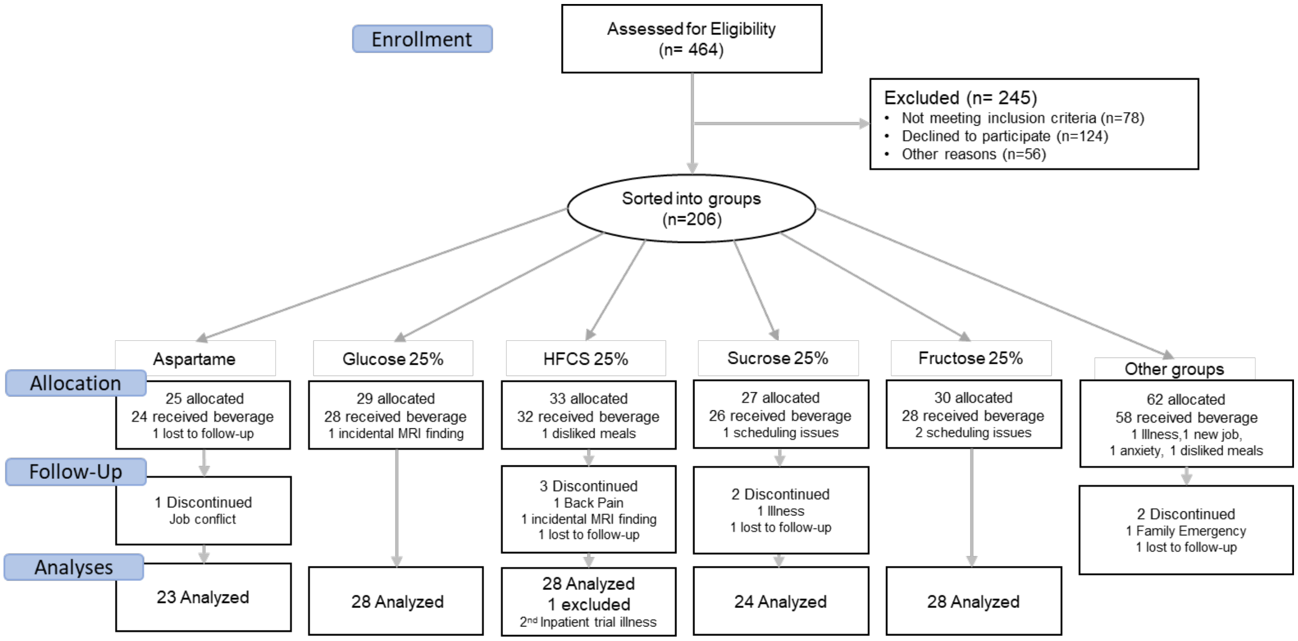

Figure S1. Number of participants allocated, lost to follow-up and included in analyses in the groups consuming aspartame-, and 25% energy requirement glucose-, HFCS-, sucrose- and fructose-SB.

S1.2 Outpatient 24-h food intake recalls

During the first 2 years of the study, estimates of food intake before and during the outpatient phase of the study were collected by 24-hour recall (via telephone) using the USDA 5-step Multiple-Pass Method as described by Conway [30]. The recalls were administered by a registered dietitian and analyzed with Nutrition Data System for Research (University of Minnesota). This method was replaced by the Automated Self-Administered (ASA) 24-h Dietary Assessment Tool (https://epi.grants.cancer.gov/asa24/) when it became apparent that the success rate for collecting the data during the pre-study period was less than 50%. The ASA Dietary Self-Administered Assessment Tool allowed subjects to enter 24-h food intake recalls using their personal computers and the computers at the CCRC. By scheduling time for the subjects to complete the ASA Dietary Assessment on the first day of each inpatient period, it was ensured a minimum of one pre-study and one outpatient intervention food intake recall would be obtained from each participant. The majority of the subjects provided only one pre-study recall and, as previously reported [23], the pre-study data was confounded by under-reporting. During the intervention period, an average of 2.3 recalls were collected from 75 participants. The dietary intake outcomes were compared between the ASA method and the Multiple-Pass methods and deemed comparable. The data were combined for a total n of 98 and analyzed via 2-factor (sugar, sex) ANCOVA with adjustment for calculated energy requirement.

S2. Results

S2.1 Body weight

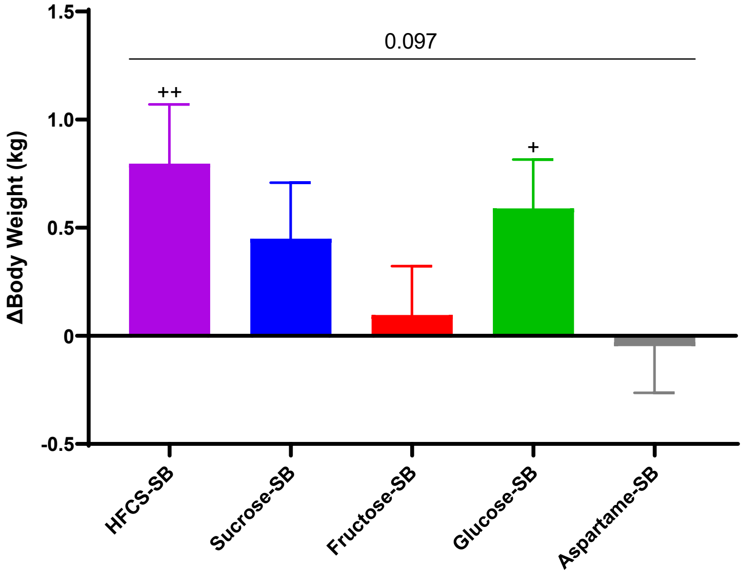

Figure S2. Δ Body weight (Week 2 – Week 0) in subjects consuming beverages sweetened with HFCS-, sucrose-, fructose-, glucose- or aspartame-sweetened beverages for 2 weeks (F). 2-factor (beverage, sex) ANCOVA with adjustment for MSRF and outcome at baseline. $^{+}P < 0.05$, $^{++}P < 0.01$, LS mean different from zero.

S2.2 Quicki

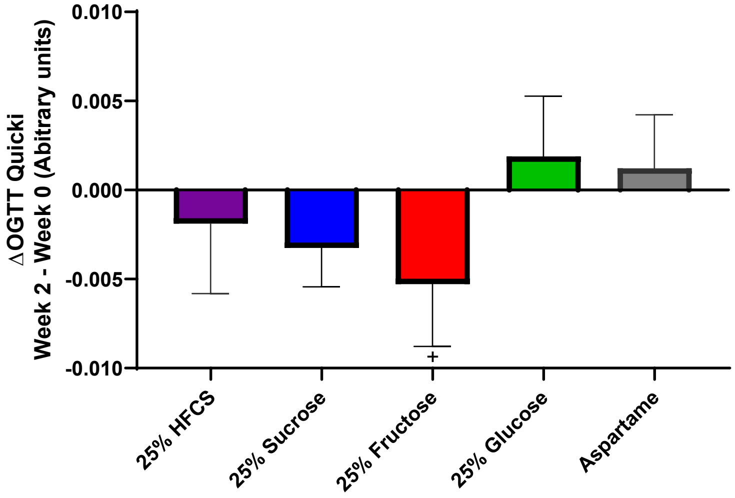

Figure S3. Δ Quicki (Week 2 – Week 0) in subjects consuming beverages sweetened with HFCS-, sucrose-, fructose-, glucose- or aspartame-sweetened beverages for 2 weeks (F). 2-factor (beverage, sex) ANCOVA with adjustment for MSRF and outcome at baseline. $^{+}P < 0.05$, $^{++}P < 0.01$, LS mean different from zero.

S2.3 24-h dietary recall

Table S1. 24-h recall energy intake during outpatient intervention

Beverage groups	HFCS-SB n = 19	Sucrose-SB n = 24	Fructose-SB n = 19	Glucose-SB n = 21	Aspartame-SB n = 15	Effect of SB[1] *P* -value
SB energy intake (kcal)	602 ± 19	588 ± 17	604 ± 20	608 ± 16	0 ± 0	...
Reported energy intake (kcal)	1,582 ± 133	1,635 ± 41	1,842 ± 151	1,713 ± 87	1,756 ± 106	0.43
Reported + SB energy intake (kcal)	2,184 ± 140^{ab}	2,223 ± 44^{a}	2,446 ± 155^{a}	2,321 ± 94^{a}	1,756 ± 106^{b}	0.0009
Energy intake/ Energy requirement[2] (%)	91.0 ± 5.3^{ab}	96.1 ± 2.9^{a}	102.2 ± 6.4^{a}	95.6 ± 3.2^{a}	77.1 ± 5.3^{b}	0.003

Values are mean ± SEM. [1]2-factor (beverage, sex) ANCOVA with adjustment for calculated energy requirement; groups without shared letters are significantly different, Tukey's post-test. [2]Calculated with Mifflin equation.

As shown in Table S1, there were no significant differences in reported energy intake among the 5 groups. However, since the subjects consuming aspartame-SB consumed intervention drinks that contained zero energy, their reported energy + SB energy intake was significantly less than the groups consuming sucrose-, fructose-, or aspartame-SB. The groups consuming aspartame-SB reported consuming only 77.1% of their calculated energy requirement, which was significantly less than that of the 4 groups who reported consuming 91-102% of calculated energy intake. Likely this 77.1% was the result of under-reporting or under-eating specific to the day prior to reporting. A 23% deficit in energy intake throughout the outpatient intervention period would have resulted in a decernible decrease in body weight of about 0.9 kg. However, the change in body weight in the group consuming aspartame-SB was -0.05 kg.

There were no group differences in the reported intakes of protein (P = 0.4), fat (P = 0.4), complex carbohydrate (P = 0.5) or sugar (excludes SB; P = 0.8) in grams. Table S2 shows the distribution of the macronutrient as % of reported energy + SB energy intake. There were no differences in macronutrient distribution among the groups consuming HFCS-, sucrose-, fructose- or glucose-SB. Compared to these group, the participants consuming aspartame reported consuming a higher distribution of protein, fat, and complex carbohydrate, and a lower distribution of total carbohydrate and total sugar.

Table S2. 24-h recall macronutrient intake during outpatient intervention

Beverage groups	**HFCS-SB** n = 19	**Sucrose-SB** n = 24	**Fructose-SB** n = 19	**Glucose-SB** n = 21	**Aspartame-SB** n = 15	Effect of SB[1] *P*-value
Protein intake/ Energy intake (%)	13.3 ± 0.6[a]	12.2 ± 0.4[a]	13.0 ± 0.8[a]	13.3 ± 0.6[a]	19.0 ± 0.5[b]	<0.0001
Fat intake/ Energy intake (%)	24.6 ± 1.3[a]	27.1 ± 0.6[a]	27.0 ± 1.6[a]	27.1 ± 1.2[a]	37.5 ± 1.7[b]	<0.0001
Total carbohydrate intake[2]/ Energy intake (%)	60.1 ± 1.5[a]	58.7 ± 0.7[a]	58.0 ± 1.8[a]	57.3 ± 1.3[a]	40.9 ± 1.8[b]	<0.0001
Complex carbohydrate intake/ Energy intake (%)	19.4 ± 1.0[a]	19.8 ± 0.7[a]	20.5 ± 0.8[a]	19.9 ± 1.0[a]	26.0 ± 1.2[b]	<0.0001
Food & milk sugar intake[3]/ Energy intake (%)	11.6 ± 1.2	12.4 ± 0.8	11.2 ± 1.1	10.7 ± 1.0	14.9 ± 1.3	0.18
Total sugar intake[4]/Energy intake (%)	40.7 ± 1.6[a]	38.9 ± 0.9[a]	37.5 ± 2.1[a]	37.5 ± 1.5[a]	14.9 ± 1.3[b]	<0.0001
Fiber/ 2000 Kcal (g)	14.0 ± 1.6	13.2 ± 1.3	13.0 ± 1.0	12.5 ± 1.0	15.6 ± 0.9	0.26

Values are mean ± SEM. [1]2-factor (beverage, sex) ANCOVA with adjustment for calculated energy requirement; groups without shared letters are significantly different, Tukey's post-test. [2]Includes complex carbohydrate and sugar from food & milk and intervention beverages. [3]Does not includes sugar from intervention beverages. [4]Includes sugar from intervention beverage. ANCOVA, analysis of covariance; HFCS, high fructose corn syrup.
